# Music medicine and music therapy in neonatal care: a scoping review of passive music listening research applications and findings on infant development and medical practice

**DOI:** 10.1186/s12887-024-05275-z

**Published:** 2024-12-23

**Authors:** Efthymios Papatzikis, Maria Agapaki, Rosari Naveena Selvan, Deanna Hanson-Abromeit, Christian Gold, Shulamit Epstein, U. Wun Vivian Lok, Evrykleia Barda, Varun Pandey

**Affiliations:** 1 Department of Early Childhood Education and Care, Oslo Metropolitan University, Oslo, Norway; 2 Advanced Health Intelligence and Brain-Inspired Technologies (ADEPT) Research Group, Oslo Metropolitan University, Oslo, Norway; 3 Department of Psychology, Metropolitan College, Athens, Greece; 4 Department for Computational Neuroscience, Third Institute of Physics – Biophysics, University of Göttingen, Göttingen, Germany; 5 Department of Psychology, University of Münster, Münster, Germany; 6 School of Music, Baby Music Intervention Research Lab, University of Kansas, Lawrence, KS USA; 7 Division of Health and Society, NORCE Norwegian Research Centre AS, Bergen, Norway; 8 Department of Clinical and Health Psychology, University of Vienna, Vienna, Austria; 9 Grieg Academy Department of Music, University of Bergen, Bergen, Norway; 10 Faculty of Psychology, University of Vienna, Vienna, Austria; 11 Independent Researcher, Athens, Greece; 12 Independent Researcher, Pune, India

**Keywords:** Passive music listening, Neonatal Intensive Care Unit (NICU), Infant development, Music therapy, Music medicine, Preterm infants, Pain management, Physiological responses, Neurodevelopment

## Abstract

**Background:**

In recent years, the use of music as a therapeutic and developmental tool for infants, especially within neonatal intensive care units (NICUs), has seen a surge in interest. Despite a growing body of research underscoring the potential benefits of music therapy and music medicine in enhancing infant development and aiding medical practices, the specific characteristics of music that maximize these benefits remain poorly understood.

**Objectives:**

This scoping review aims to provide a comprehensive foundation by mapping the existing literature on passive music listening and identifying gaps, trends, and patterns that are crucial precursors to the development of best practices.

**Methods:**

Following the PRISMA guidelines, a comprehensive literature search was conducted, covering studies published up until December 2022. The focus was on passive music listening, with a deliberate exclusion of active music interventions. Out of the initial pool of studies, 56 met the inclusion criteria, determined by the PICO framework, focusing on passive music exposure among full-term and preterm infants. Starting with a descriptive analysis approach, the study employed Non-negative Matrix Factorization (NMF) and Latent Dirichlet Allocation (LDA) to identify key themes, including the physiological impacts of music, its role in pain management, effects on sleep and stress, and influences on feeding and weight gain.

**Results:**

The review revealed a predominance of quantitative research methods, a significant concentration of studies from the United States, suggesting potential geographical bias, and a notable clinical setting bias. These findings indicate a critical need for methodological diversity and a more culturally inclusive and interdisciplinary approach to research. They also point to the necessity for standardized music intervention protocols to optimize therapeutic and developmental outcomes for this vulnerable population. Future research should aim to bridge the methodological gaps identified, integrating both qualitative and quantitative approaches to provide a comprehensive understanding of music’s impact on infant development and medical practices in a global, culturally nuanced context.

**Supplementary Information:**

The online version contains supplementary material available at 10.1186/s12887-024-05275-z.

## Introduction

Recent years have witnessed a surge in interest concerning the employment of music within neonatal intensive care units (NICUs). This increasing attention stems from a multitude of studies establishing the potential advantages of music therapy and music medicine for infant development, as well as its contributions to medical practices. Music therapy is defined as a clinical and evidence-based use of music interventions to accomplish individualized goals within a therapeutic relationship by a credentialed professional who has completed an approved music therapy program [46, 47]. In contrast, music medicine refers to the use of music in medical settings without the involvement of a qualified music therapist, often through the use of recorded music, and is not limited to the presence of a therapist-client relationship [47].

Remarkably, infants exhibit a heightened responsiveness to auditory stimuli, which is attributed to their auditory system’s early maturation. Even before birth, they can perceive and process complex auditory inputs [29, 45], and this precocious auditory sensitivity is thought to play a pivotal role in the subsequent development of an array of cognitive and socio-emotional skills. Studies on passive music listening have shown improvements in physiological responses and sleep [6, 11, 26, 37], while research on active music engagement has demonstrated benefits in cognitive, motor, and social-emotional development [3, 5, 13, 43, 14, 21]. Additionally, active musical participation has been shown to bolster motor skills, coordination and rhythm synchronization, by furnishing a structured, temporally organized context for movement [17, 18, 30, 36].

Music, and lullabies more specifically, have been shown to foster social-emotional development in infants. Active musical engagement in this framework, involving singing and musical interaction with caregivers, can enhance attachment and bonding while also promoting empathy, emotional regulation, and social communication skills [22, 40, 42]. Furthermore, the calming and soothing effects of passively listening to lullabies are well-established in both premature and healthy infants, shown to impact medical practices, including pain management, stress reduction, and sleep promotion [4, 6, 11, 20, 26, 35, 38, 39].

Yet, the complexity of music’s impact on infant development, especially in preterm infants, remains an area fraught with ambiguities and methodological discrepancies. This is starkly highlighted in the recent Cochrane review by Haslbeck et al. [16] which analyzed 25 trials - showcasing both active and passive engagement with music - with over 1500 infants and parents in NICUs. Their findings paint a picture of inconsistent outcomes and methodological diversity, failing to establish a clear consensus on the efficacy of music therapy and music medicine in this context. Specifically, they found no significant improvements in oxygen saturation or developmental scores and only minimal impact on parental anxiety, contrasting with the expected benefits often highlighted in literature. Although a reduction in heart rates of infants was indeed observed, this singular positive outcome amidst a sea of ambiguous or non-significant results calls into question the overall effectiveness and application of such interventions in NICUs.

As a result, one overarching observation in this field of research is the significant knowledge gap regarding the specific characteristics of music that optimize its developmental and medical benefits. This gap is particularly relevant when designing music delivery protocols, especially in neonatal intensive care units (NICUs), where passive listening - as opposed to active listening [see examples in 12, 13, 19, 32; further details on this important dichotomy are provided below] - may serve as a crucial intervention for vulnerable infants. This gap, as evidenced by the findings of Haslbeck et al. [15], leads to the current study’s endeavor to conduct a literature scoping review on the framework and impact of passive music listening on infant development and medical practice.

Nevertheless, while previous reviews [for example, please see 4, 10] have predominantly focused on ‘how’ music influences these areas, the present study aims to extend this understanding by addressing the ‘whats’ of the existing literature. This involves a comprehensive examination of what is currently known and mostly practiced at a technical and research design level. For example, what are the groups of infants that are mostly studied, what are the dominant methodological trends and analytical techniques used in these studies, what are the major settings and lengths of these studies, what interdisciplinary approaches are involved, what key themes and patterns exist across these studies, and what areas are perhaps under-researched or lack sufficient data, to name just a few. Such an approach is critical for building a complete picture of the field, identifying the strengths and limitations of current knowledge, helping us to finally explore in a more comprehensive and practical way the generic research question: ‘How does passive listening of music influence medical practices and development in infants, both premature and full-term’?

By focusing on answering this question through the aforementioned angle, this study aspires to contribute to a more nuanced understanding of the role of passive music listening in infant care, aims to bridge the gap between theoretical knowledge and practical application through a different point of access to evidence, while guiding future research and clinical practices to optimize the use of both music therapy and music medicine as developmental and therapeutic tools for infants. The objective is to provide actionable insights that can inform policies and protocols in this very sensitive yet extremely interdisciplinary context, ultimately benefiting this vulnerable population in a more informed and effective manner.

## Why passive music listening?

This review specifically concentrates on passive music listening in infant development and medical practice. For that matter, we approach passive listening as entailing infants being exposed to calming, soothing music or lullabies without active engagement or interaction with musical stimuli or caregivers. This form of listening primarily engages the auditory system, in contrast to active listening, which involves direct engagement with musical stimuli, often through interactive activities with caregivers or participation in musical games or instruments or engaging multiple sensory pathways including tactile and visual senses. In the context of neurodevelopmental impact, the study by Remijn and Kojima [32] sheds light on the distinct neural processing involved in passive versus active listening. This contrast between passive and active music listening modes is essential to understand, as they may influence infant development via separate neural pathways and cognitive processes. Particularly for preterm infants in Neonatal Intensive Care Units (NICUs), where active engagement is not always possible, passive music listening may serve as the only practical approach for providing auditory enrichment, while offering a soothing and stabilizing effect for infants in resting states or during periods of restricted caregiver interaction.

## Methodology

### Literature review design

In compliance with the PRISMA (Preferred Reporting Items for Systematic Reviews and Meta-Analyses) guidelines [25] as well as the PRISMA-ScR checklist, we conducted a comprehensive and systematic literature search. Our search covered the Google Scholar database for pertinent articles published up until December 2022. We opted to use only Google Scholar for our scoping review due to its strengths in providing comprehensive cross-disciplinary results and expansive coverage of both scientific journals and gray literature. This approach proved to be particularly suitable for our topic, which intersects with various fields including music therapy, music medicine, clinical care, neonatology, neurology and music, ensuring we did not overlook any relevant studies that might not be indexed in more specialized databases like PubMed. Furthermore, we also manually checked three published systematic reviews [24, 31, 41] which were close or related to the domain pertaining to our study.

In our search approach, we employed a combination of search terms using Boolean operators to ensure comprehensive coverage. The following search strings (in various sequences) were used:

- Search string 1: (“lullaby” OR “music”) AND (“brain” OR “development”) AND (“infant” OR “crib” OR “cradle”).
- Search string 2: (“lullaby” OR “music”) AND (“clinical” OR “neurology”) AND (“infant” OR “pediatrics”).

These broader search terms were chosen intentionally to capture relevant information from more focused context or framework areas, such as ‘music therapy’, ‘music medicine’, ‘pediatrics’, or ‘clinical’, without restricting our review scope. No particular filters (e.g., date, language) were applied during the search process, ensuring a wide net was cast over the literature. Additionally, to maintain thoroughness, we screened the first 100 pages of search results for each search string. This extensive review allowed us to capture relevant studies that may appear lower in the search rankings due to the nature of the platform’s indexing.

In order to qualify for inclusion in this scoping review, studies had to meet the following PICO framework criteria:

- *Populations*: full-term and/or preterm infants (birth to 12 months; corrected or not age when premature).
- *Interventions*: passive listening of music or lullabies.
- *Comparisons*: not applicable.
- *Outcomes*: medical treatment, biological development and/or clinical contexts.

Furthermore, studies had to be in English and published following a peer-reviewed procedure, while all of them had to be original studies (Randomized/Non-randomized). We excluded studies based on the following exclusion criteria:

- *Mutli-modal Interventions*: Studies employing multi-modal or multisensory protocols offer information on interventions involving multiple strategies blended with music, making it difficult to isolate the effects of music from other sensory applications.
- *Outcomes*: Studies primarily centered on cultural, cognitive, or musicological aspects of infant music exposure.
- *Study types*: Review articles or meta-analyses were not included in the analysis, but reference lists of such reviews were examined for potentially relevant original studies.

### Study selection

Two independent reviewers, who later became co-authors of this study, were tasked with screening the titles and abstracts of the retrieved articles using the aforementioned eligibility criteria. The same reviewers then assessed the full-text articles that satisfied the initial screening criteria for eligibility. In cases where disagreements arose between the reviewers, a consensus was reached through discussion, or by consulting a third reviewer, if necessary.

### Study sample

**Fig. 1:**
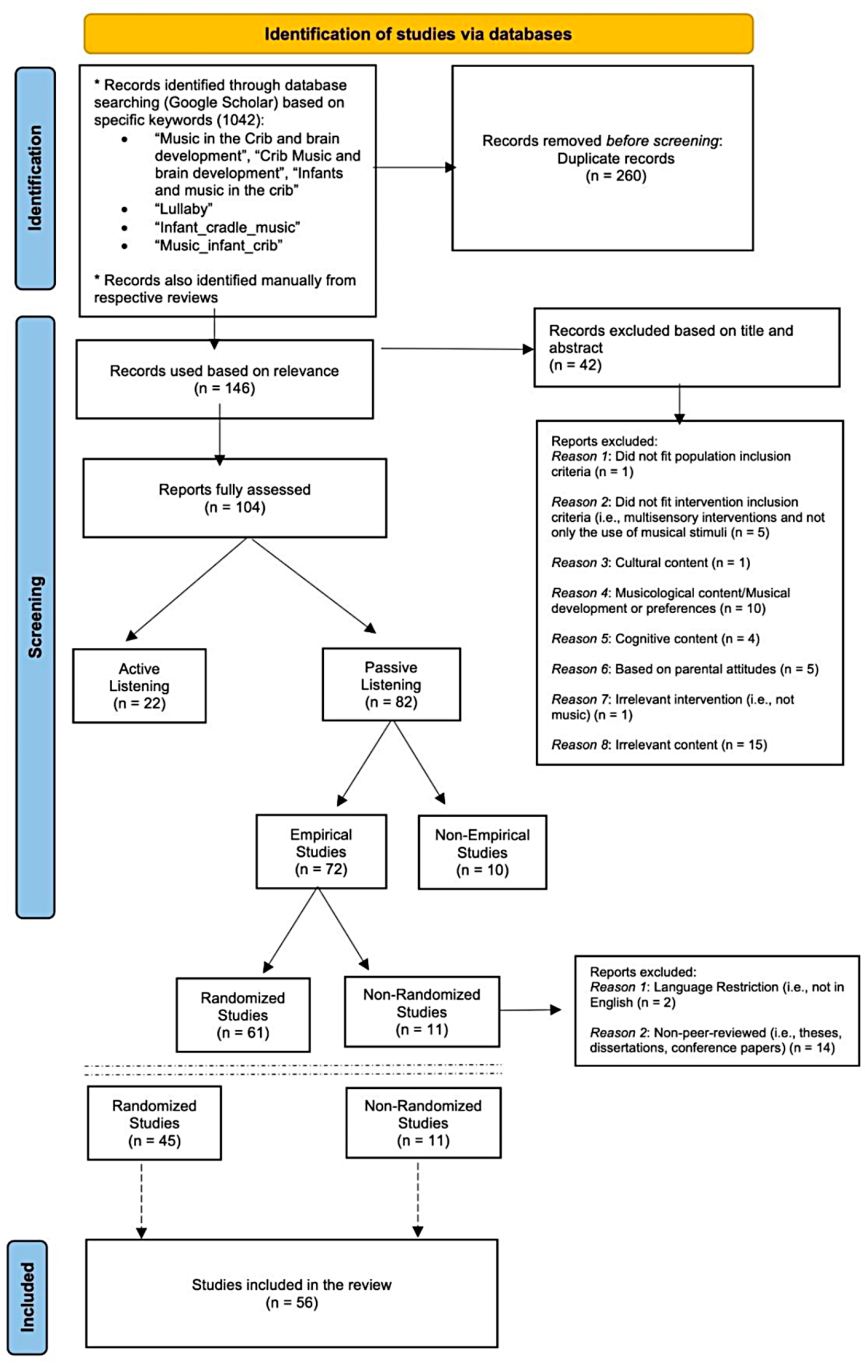
Flow diagram of the study selection process

The initial search identified a total of 1,042 articles. After removing duplicates and checking for relevance, 146 articles remained for an initial abstract screening. Following this initial screening, 104 articles were considered for full-text review. Of these, 56 articles met the eligibility criteria and were finally included in the review. A flow diagram of the study selection process is presented in Fig. 1 (see also supplementary files SF1 and SF2 for a full list of the studies included).

### Data extraction

Before beginning the data extraction process, two custom Excel spreadsheets were designed to serve as data extraction tools. The first spreadsheet featured 23 primary headings, each divided into multiple subcategories [see supplementary file SF1 for specifics], while the second spreadsheet was designed to catalog the key findings, strengths, and limitations of each one of the 56 studies [see supplementary file SF2 for specifics]. In the first spreadsheet, the headings were carefully selected to capture a broad range of variables including bibliographic details, research methodologies, analytical approaches, and demographic information. With this tool, the following details were extracted and further used for a descriptive statistics analysis:

- Bibliographic Information: This included the title, number of authors, country of origin, and publication year.
- Research Focus and Context: The primary knowledge context (Development, Medical/Treatment/Therapy, Prevention) was identified.
- Methodology and Quality: The research methods employed were categorized as qualitative, quantitative, or mixed. Information on the validity and reliability of these methods was also noted.
- Analytical Techniques: The types of qualitative and quantitative analyses used were extracted.
- Data Collection: Information about how the data were collected and which tools were used was documented.
- Sample Characteristics: This included the population focus (term newborns, preterm newborns, infants) and any pathological conditions mentioned.
- Research Context: The setting in which the research was conducted was noted, such as a hospital, nursery, or domestic environment.
- Length of Study: This was captured to understand the time frame over which observations were made.
- Interdisciplinary Involvement: Any involvement from other disciplines or professions was extracted.
- Gender and Sex Orientation: Information was extracted on whether the study separated data by biological sex of the participants’ sample.

To ensure the reliability of the data extraction process, a ‘double extraction’ method was employed, where two independent researchers extracted data from the same article. Any discrepancies were resolved through discussion or consultation with a third researcher.

Furthermore, to understand more deeply the content of our study sample, a systematic analysis involving just the findings of the 56 included studies was undertaken. Using the second spreadsheet as our data extraction tool, we performed a meticulous review of each article to export and organize their findings individually. This spreadsheet served as a foundational dataset, on which a subsequent Non-negative Matrix Factorization (NMF) was performed (using a custom-made Python code), as well as a Latent Dirichlet Allocation (LDA) for topic modeling and in-depth analysis of emerging themes and patterns within the specific research landscape.

The choice of NMF was motivated by the several advantages it offers, including its capacity to generate interpretable topics, computational simplicity, and sparse representation. NMF allows for a minimal number of topics to describe each available dataset, facilitating easier interpretation and meaningful topic discovery. On the other hand, LDA, while simpler and less powerful than NMF, played a complementary role in our text analysis. It served to cross-reference the results obtained from NMF, ultimately providing an averaged computation of the most prominently emerging topics within the findings of the 56 reviewed studies.

The NMF technique involved the conversion of the textual data into a numerical representation using Term Frequency-Inverse Document Frequency (TF-IDF) vectorization. The TF-IDF is a numerical statistic used in text mining and information retrieval to reflect how important a word is to a document in a collection or corpus of documents (dataset). It is a way to weigh the importance of terms (words) in the document based on how frequently they appear across all documents. The TF-IDF was calculated with the following equation:

$$\:\text{TF-IDF(t,\:d,\:D)\hspace{0.17em}=\hspace{0.17em}TF(t,\:d)\:x\:IDF(t,\:D)}$$

Where t = Number of times term t appears in document/text d.

Where d = Total number of terms in document/text d.

Where D = Total number of documents/text in corpus D.

After the TF-IDF matrix was generated (see SF3), it was then decomposed into two lower-dimensional matrices, *W* (see SF4) and *H* (see SF5); where *W* represents the “document-topic” relationship and *H* encapsulates the “topic-word” relationship. The *H* matrix was examined to identify the top words that are representative of each topic, and then each “document” (in our case, each study’s key findings) was assigned to the topic for which it had the highest representation in the *W* matrix.

$$\:\text{TF-IDF\:Matrix\hspace{0.17em}=\hspace{0.17em}W\:x\:H}$$

In contrast to the TF-IDF-based approach used for NMF, LDA operates by modeling documents as mixtures of topics, where each topic is characterized by a distribution of words. In our LDA analysis, the textual data underwent preprocessing, including tokenization and stop word removal. LDA then inferred the underlying topics within the corpus of documents (dataset) and the distribution of words associated with each topic. This process involves a probabilistic model that assigns words in each document to specific topics, and topics to documents, aiming to find a coherent and meaningful representation of the content. After running LDA, we obtained a set of topics and their associated word distributions. These topics served as a way to categorize and summarize the content of the reviewed studies. For each topic, we identified the most representative words, offering insights into the key themes present in the dataset.

Following the completion of both NMF and LDA analyses and the identification of primary topics within the key findings of the 56 studies, Principal Component Analysis (PCA) emerged as a final yet critical step in our research methodology. The utilization of PCA served several pivotal functions in enhancing our understanding of the findings dataset and further refining our analytical approach. Initially, NMF and LDA provided valuable insights into the specific dataset by identifying key topics and recurring themes. However, the inherent complexity of the dataset, consisting of numerous variables and topics, required a means of dimensionality reduction to simplify interpretation. This necessity led us to incorporate PCA into our analysis. In that sense, PCA effectively reduced the dimensionality of the high-dimensional dataset, while this transformation facilitated a more concise and comprehensible representation of our dataset, enabling us to delve deeper into the underlying patterns of the findings. As PCA allowed us to visualize the intricate relationships and dependencies between topics and variables, it provided a visual context that was complementary to the results obtained from NMF and LDA.

## Results

### Descriptive statistics

**Fig. 2:**
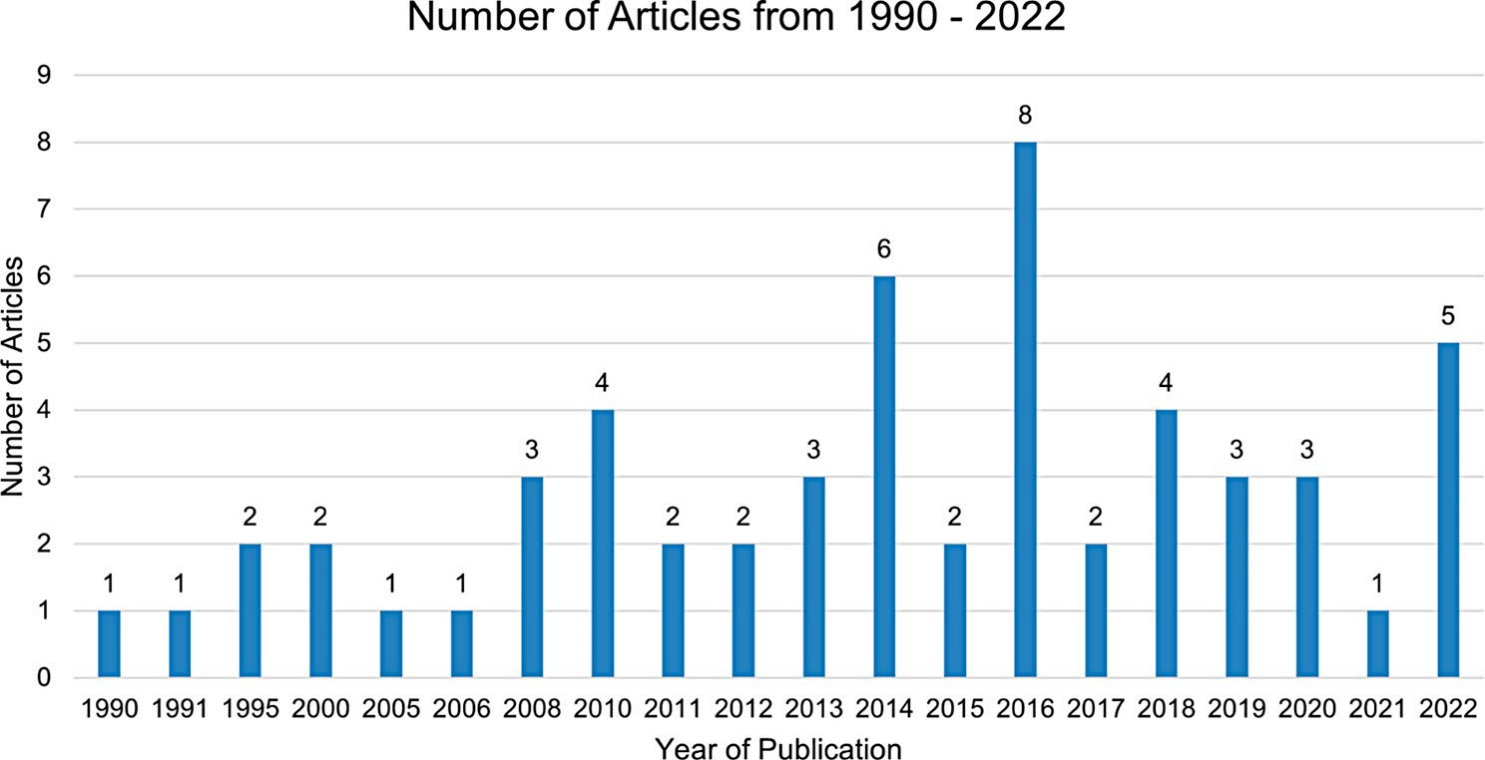
Studies conducted between 1990 and 2022 per year

**Fig. 3:**
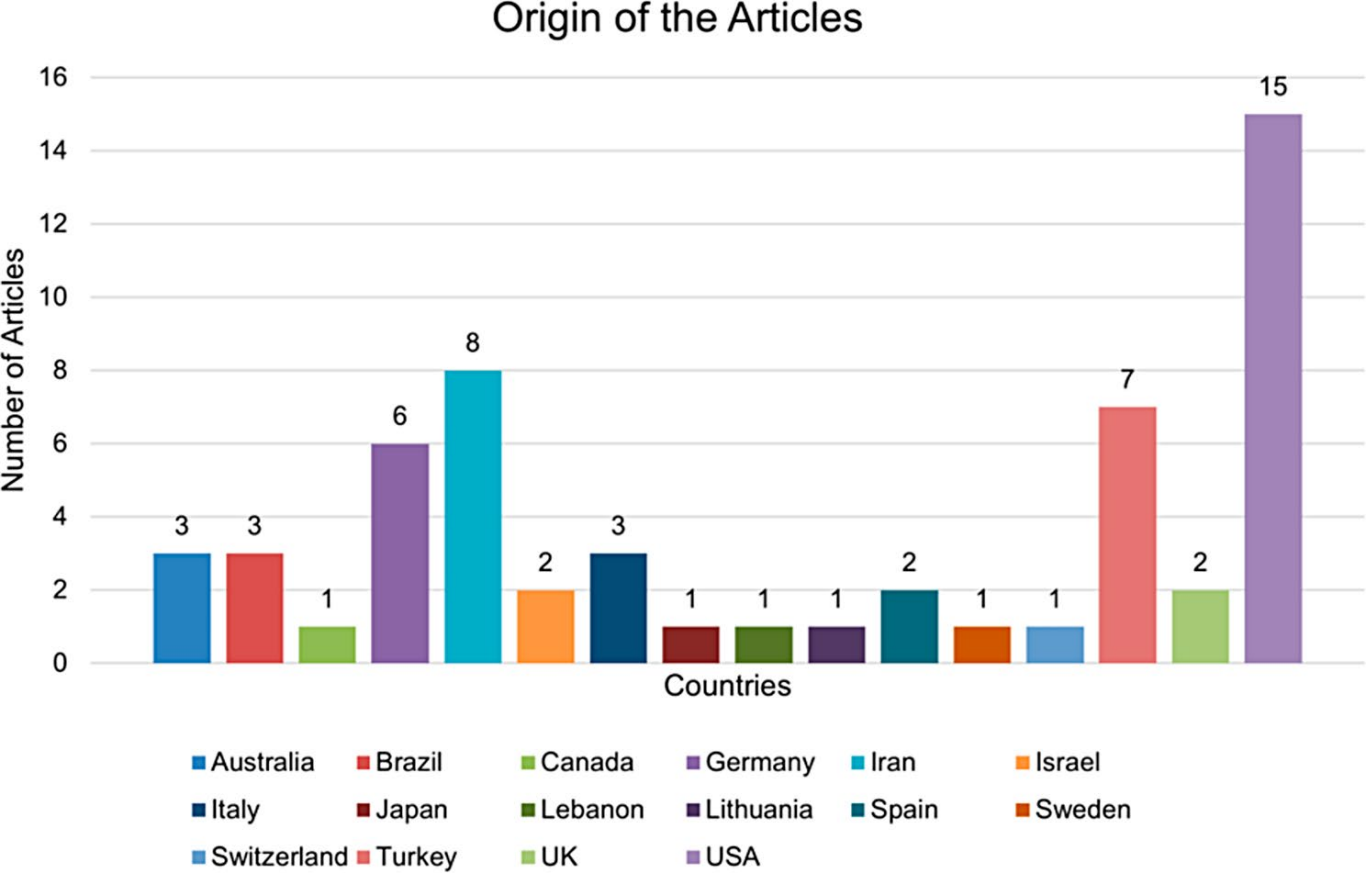
Geographical origin of the studies carried out between 1990 and 2022

**Fig. 4:**
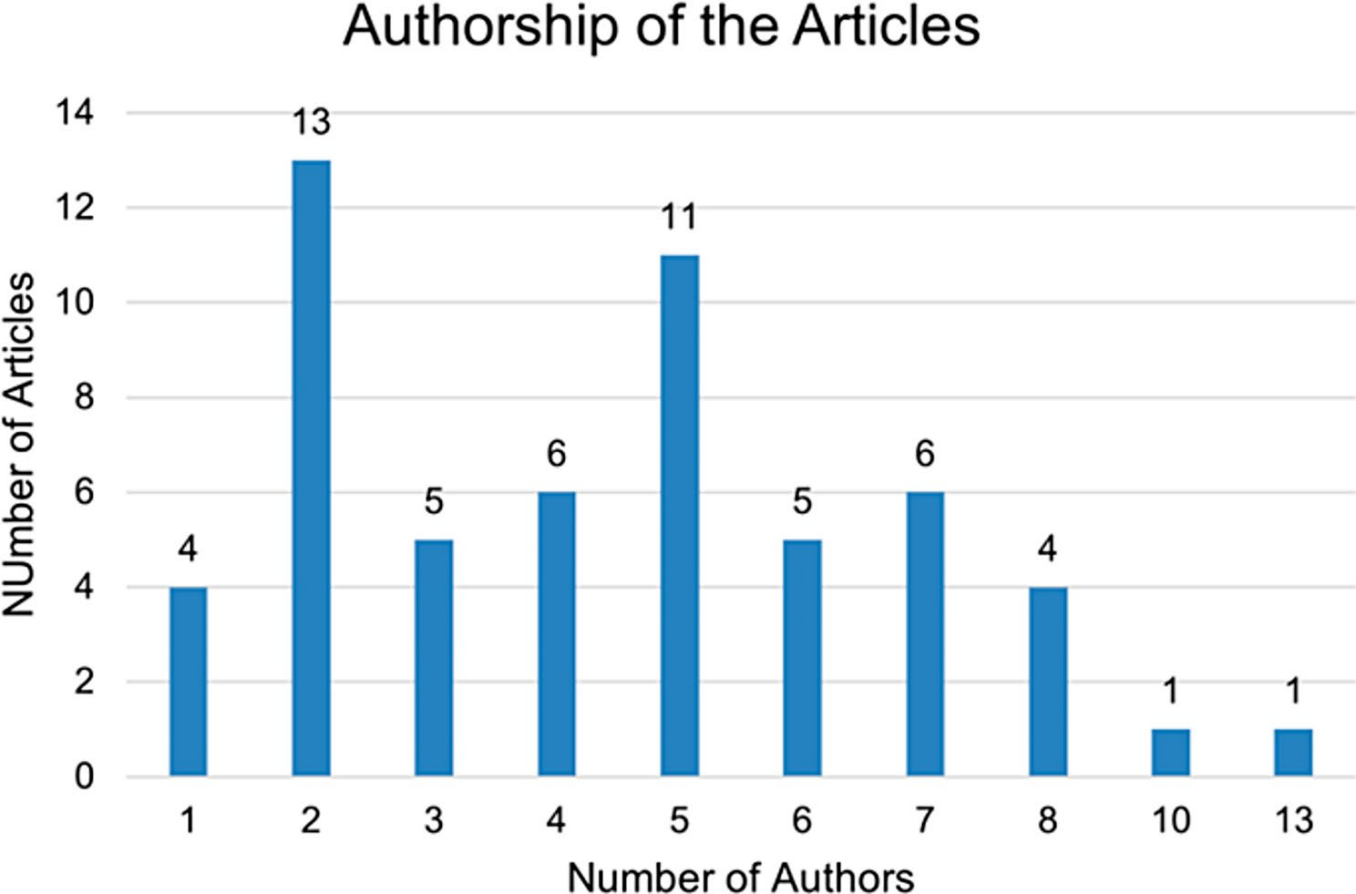
Contributing authors per article

Our analysis rigorously assessed the extensive collection of studies carried out between 1990 and 2022 (Fig. 2). Though the studies were geographically diverse, a significant portion originated from the United States (Fig. 3). In terms of authorship, the studies exhibit considerable variation, with the number of contributing authors ranging from a minimum of one to a maximum of 13 (Fig. 4).

### Thematic analysis of titles

An analysis of the study titles - using a custom-made python script - revealed the most researched broader themes (Table 1), which included the influence of mothers, indicated by words such as “mothers” and “maternal”; the timing of interventions or observations, represented by the word “during”; and the use of recorded music in studies, implied by the term “recorded”. Other focal points were pain management, physiological focus, therapeutic methods, and a comparison between live and recorded interventions or stimuli, highlighted by words like “pain”, “physiological”, “responses”, “therapy”, “live”, and “singing”. This thematic analysis was derived from a word frequency count on the study titles, excluding common English words like “the”, “and”, “of”, “in”, and “to”, which do not contribute to the thematic content.

**Table 1:** Frequency of the most researched broader themes in study titles

Theme Word	Frequency
mothers	8
during	8
recorded	8
pain	8
physiological	7
responses	7
therapy	6
live	6
maternal	5
singing	5

Further, a co-occurrence analysis of theme-related words in the study titles brought to light interesting relationships and potential focal points in the research landscape. For instance, the terms “maternal” and “singing” co-occurred four times, suggesting a significant focus on the influence of maternal singing on various outcomes, possibly including infant development or psychological well-being (see Table 2). Similarly, the pairing of “physiological” and “responses,” appearing together four times, indicated a frequent exploration of physiological responses to different stimuli or conditions. The analysis unveiled several other notable co-occurrences, such as “during” and “singing” and “during” and “pain”, each appearing three times, hinting at studies focusing on real-time effects of singing and pain management techniques during specific procedures or behavior states, respectively. This detailed co-occurrence analysis not only helped us underscore the interrelated themes in the dataset but also pointed out a quite good profile of diversity of research avenues explored in the studies, ranging from the therapeutic effects of singing to the comparative analysis of live versus recorded stimuli.

**Table 2:** A co-occurrence analysis of theme-related words in the study titles

Co-occurring Themes	Frequency
(‘maternal’, ‘singing’)	4
(‘physiological’, ‘responses’)	4
(‘during’, ‘singing’)	3
(‘during’, ‘pain’)	3
(‘during’, ‘maternal’)	2
(‘maternal’, ‘recorded’)	2
(‘live’, ‘recorded’)	2
(‘live’, ‘physiological’)	2
(‘singing’, ‘therapy’)	2
(‘physiological’, ‘recorded’)	1
(‘recorded’, ‘responses’)	1
(‘live’, ‘responses’)	1
(‘recorded’, ‘therapy’)	1
(‘recorded’, ‘singing’)	1
(‘maternal’, ‘therapy’)	1
(‘during’, ‘physiological’)	1
(‘pain’, ‘physiological’)	1
(‘during’, ‘therapy’)	1
(‘during’, ‘live’)	1
(‘live’, ‘therapy’)	1
(‘live’, ‘singing’)	1
(‘during’, ‘mothers’)	1
(‘mothers’, ‘pain’)	1
(‘mothers’, ‘physiological’)	1
(‘live’, ‘mothers’)	1
(‘maternal’, ‘responses’)	1
(‘responses’, ‘singing’)	1

### Quantitative vs. qualitative approach

**Fig. 5:**
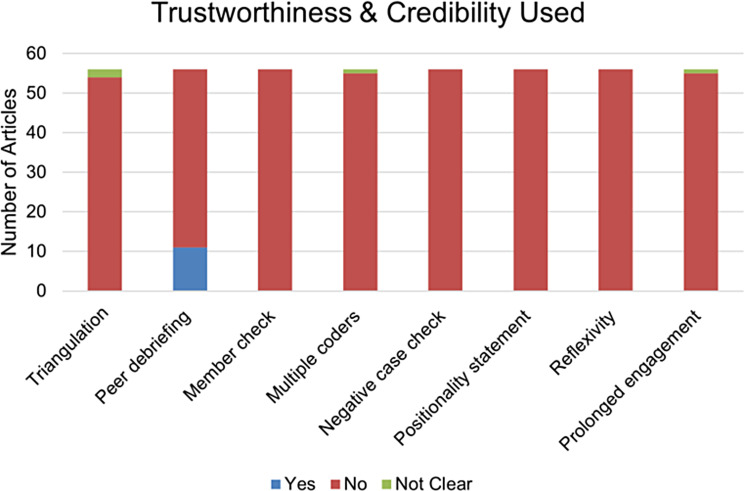
Relationship between reported trustworthiness and existing credibility measures in qualitative methods

**Fig. 6:**
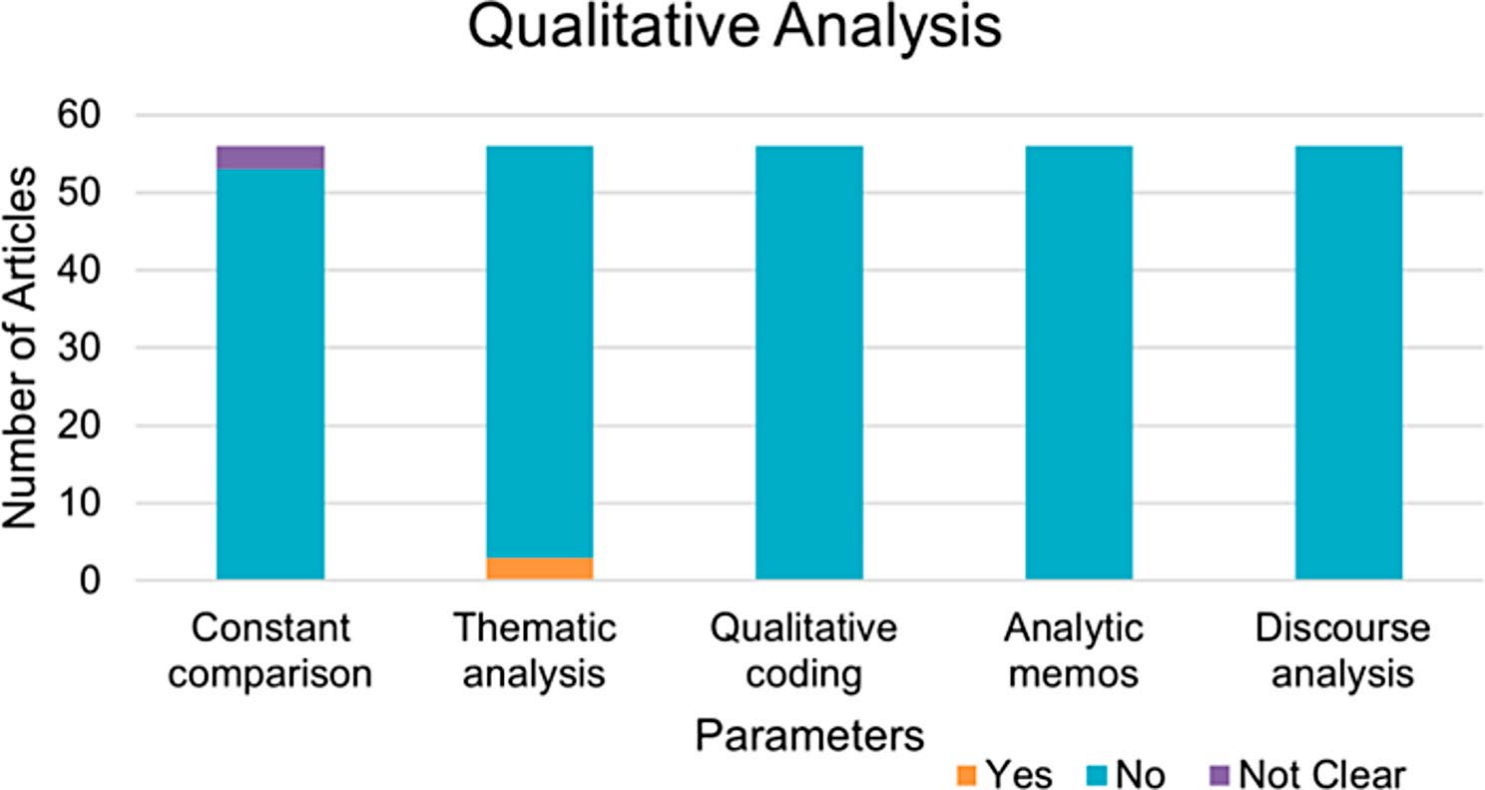
Parameters evaluated for the process of examining, interpreting, and understanding qualitative data

Out of the 56 studies analyzed, a significant portion adopted quantitative approaches (82%), while a smaller number were based on mixed (16%) and qualitative (2%) methodologies. Especially for the qualitative methods, we found a significant Chi-Square statistic (χ^2^ = 29.42) with a very low p-value (*p* = 0.0064) when examining the relationship between reported trustworthiness and existing credibility measures in qualitative methods (Fig. 5). Furthermore, we found that the process of examining, interpreting and understanding non-numerical data collected during the studies were tested using five parameters namely constant comparison, thematic analysis, qualitative coding, analytic memos and discourse analysis (Fig. 6).

**Fig. 7:**
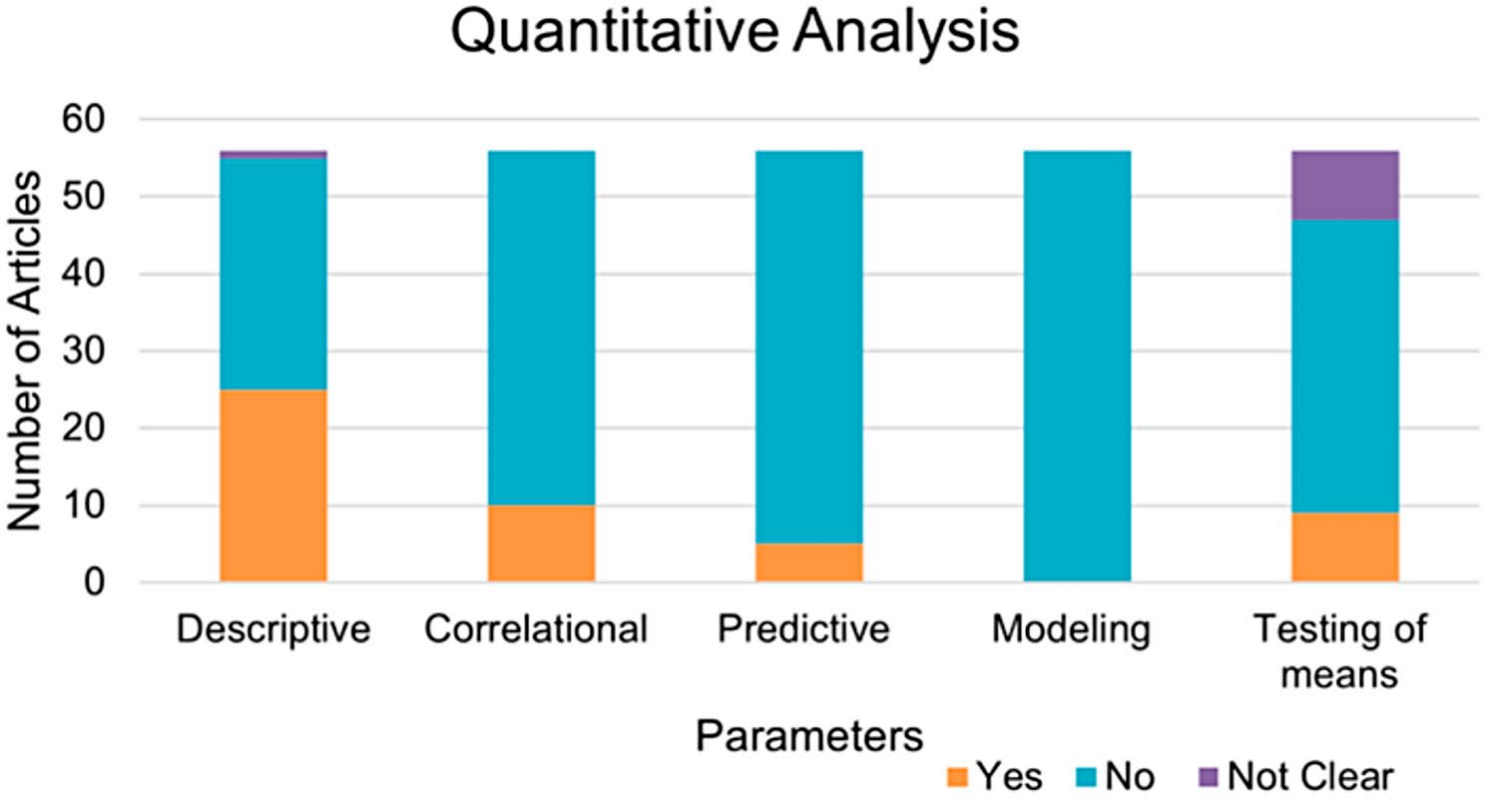
Statistical analyses used for the process of examining, interpreting, and understanding quantitative data

On the other hand, for the quantitative methods, their statistical analyses predominantly adopted descriptive, testing of means, and correlational approaches (Fig. 7) revealing a troubling lack of diversity. This was substantiated by a significant Chi-Square statistic (χ^2^ = 56.0) and an extremely low p-value (*p* = 0.007) shown after a correlational analysis of validity measures within these quantitative methods.

### Research contexts

**Fig. 8:**
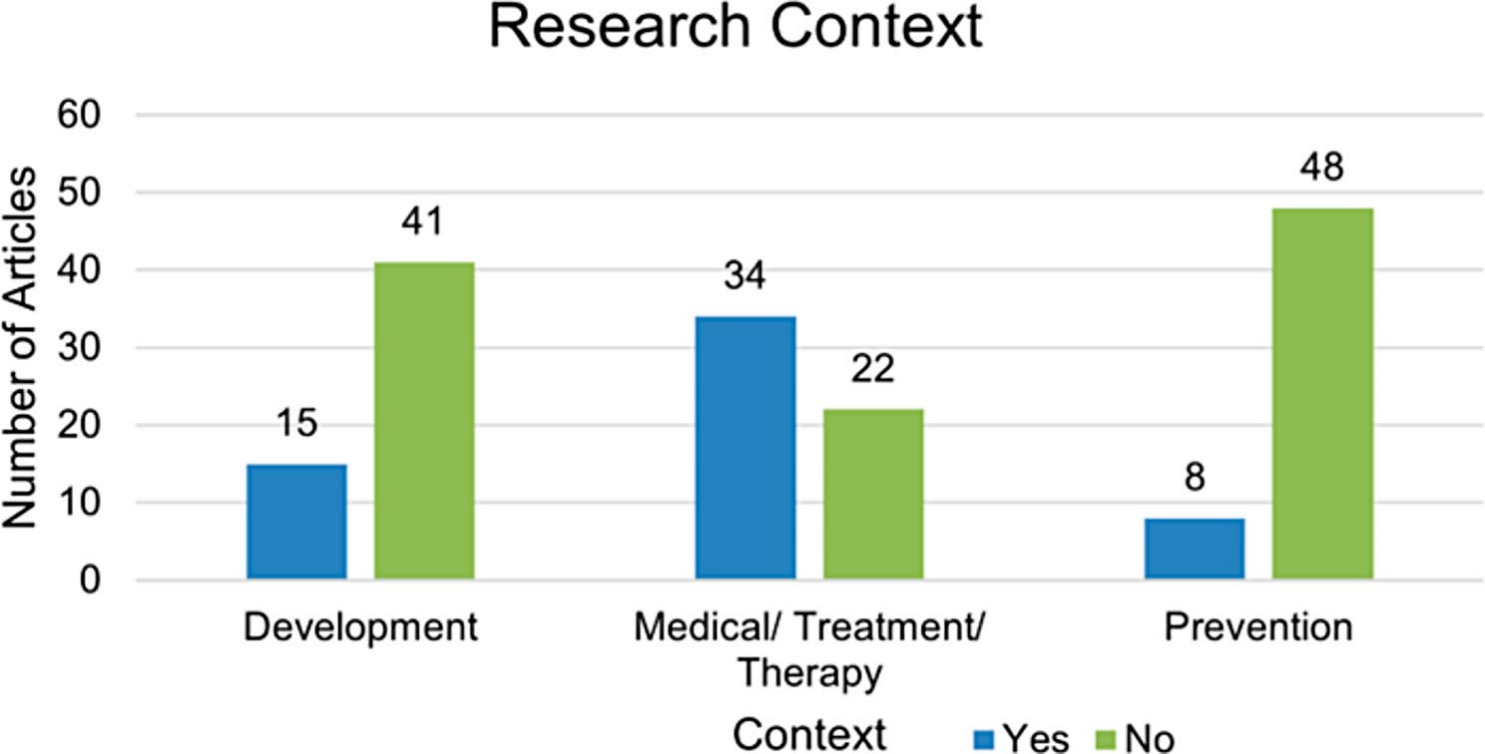
Research contexts used in selected studies

In the corpus of research studies analyzed, a predominant focus was observed on the application of music in medical treatment and therapeutic interventions, overshadowing its utilization in developmental and preventative contexts (Fig. 8).

### Data collection methods

Our dataset revealed a diverse range of methods for data collection in these studies, encompassing physiological measures such as Heart Rate (HR), Oxygen Saturation, and Respiratory Rate (RR), clinical assessment tools including the APGAR score and PIPP scale, and various questionnaires. Advanced neuroimaging techniques such as EEG, fMRI and LC-MS (Liquid Chromatography–Mass Spectrometry) were also utilized, alongside observation methods like interviews and video recordings. In total, there were 54 unique methods of data collection, each used in one study, except for fMRI and the PAL device, which were employed in two studies each.

### Populations involved

In examining the populations studied, most studies focused on term newborns (11 studies) and preterm newborns (47 studies) either individually or in combination. Studies mentioning infants only - as a generic code - as their respective studied population were very rare, appearing only in one of the studies.

### Setting of research

A striking 96% of the studies within this research domain were conducted in hospital settings, highlighting the field’s pronounced emphasis on clinical contexts. Conversely, non-clinical settings such as domestic environments and rehabilitation centers were significantly under-represented, constituting only 4% and 2% of the 56 studies, respectively. It should be noted that the cumulative percentages may exceed 100% due to the inclusion of multiple settings within individual studies.

### Biological sex

**Fig. 9:**
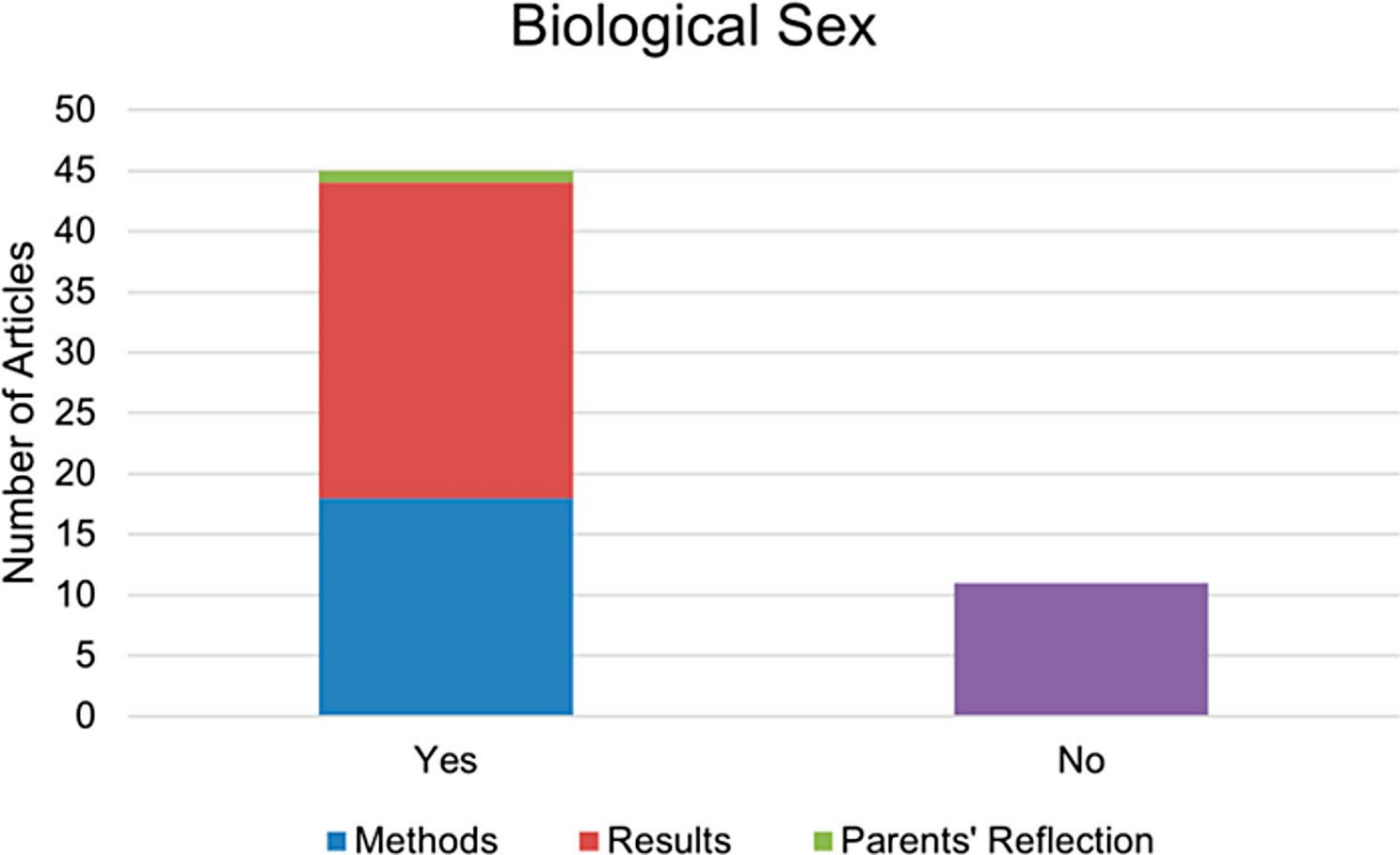
The presence or absence of methodological differentiation based on Biological sex

The field of biological sex has adopted a varied approach; some studies have refrained from differentiating based on sex, while others have concentrated exclusively on one sex or have established distinct groups for male and female participants. The following figure (Fig. 9) encapsulates the outcomes of our analysis with respect to the presence or absence of methodological differentiation based on sex. A total of 18 studies explicitly mentioned sex separation in their methodology, while 26 studies did not mention sex differentiation in the methodological section - yet they revealed such differentiation during the data analysis phase. Notably, one study utilized parents’ reflections as a method to ascertain the sex of the subjects. In the remaining 11 studies, sex separation was neither mentioned nor implemented.

### Research tools

In our scrutiny of the 56 studies, we noted the utilization of a broad spectrum of research tools employed across different methodologies and data collection strategies. These tools can be categorized into the types shown in the following table (Table 3):

**Table 3:** Research tools and their descriptions used in various techniques and data collection strategies

Category	Description
Audio Equipment	Includes micro-audio systems, speakers, headphones, and MP3 players.
Video Equipment	Various models of digital video cameras were used.
Medical Research Devices	Devices such as pulse oximeters, decibel meters, and thermal analyzers were common.
Tools for Clinical Analysis	These encompassed specialized software and devices for data analysis like ELISA, ActiGraph, and HRV Scanner.
Miscellaneous	This category captured custom-made tools, musical instruments, and other specialized equipment employed for unique study requirements.

### Representation of professionals

Our analysis revealed a conspicuous lack of detail in depicting the range of professionals engaged in the 56 studies. These ranged from nurses and music therapists to audiologists and academic researchers, including both codes of “chief investigator” and “nurse researchers”. Importantly, when we examined the correlation between the disciplines or professions involved and the countries of origin, our statistical analysis yielded a strikingly high Chi-Square statistic (χ^2^ = 577.3) coupled with a remarkably low p-value (*p* = 0.003). This significant result suggests a strong association between the professional roles involved in these studies and the countries where the research was conducted. The high Chi-Square statistic indicates that the observed distribution of professionals across different countries deviates substantially from what would be expected by chance alone (i.e., very low p-value). In other words, certain countries appear to favor the involvement of specific types of professionals in research, which may reflect underlying cultural, systemic, or institutional factors.

### Journals-pathology-length of intervention

In our comprehensive examination of the 56 studies, several journals emerged as dominant platforms for research in this specific domain. Notably, *Acta Paediatrica* and the *Journal of Music Therapy* each accounted for five publications, while the *Journal of Neonatal-Perinatal Medicine*,* Pain Management Nursing*, and *Journal of Perinatology* each contributed two publications. These figures highlight the significant role these journals play in shaping academic discourse and advancing research in the field.

Turning to the issue of pathology, an analysis of the value distribution in our dataset points to an intriguing pattern. In 34 out of 56 studies, no pathology was explicitly mentioned, comprising approximately 61% of the research corpus. On the other hand, 22 studies did mention pathology, making up around 39% of the studies.

Lastly, concerning the length of interventions, it is worth noting that this crucial aspect remains largely underreported. Out of the 56 studies, only two explicitly indicate the length of the interventions undertaken.

## Findings analysis

**Fig. 10:**
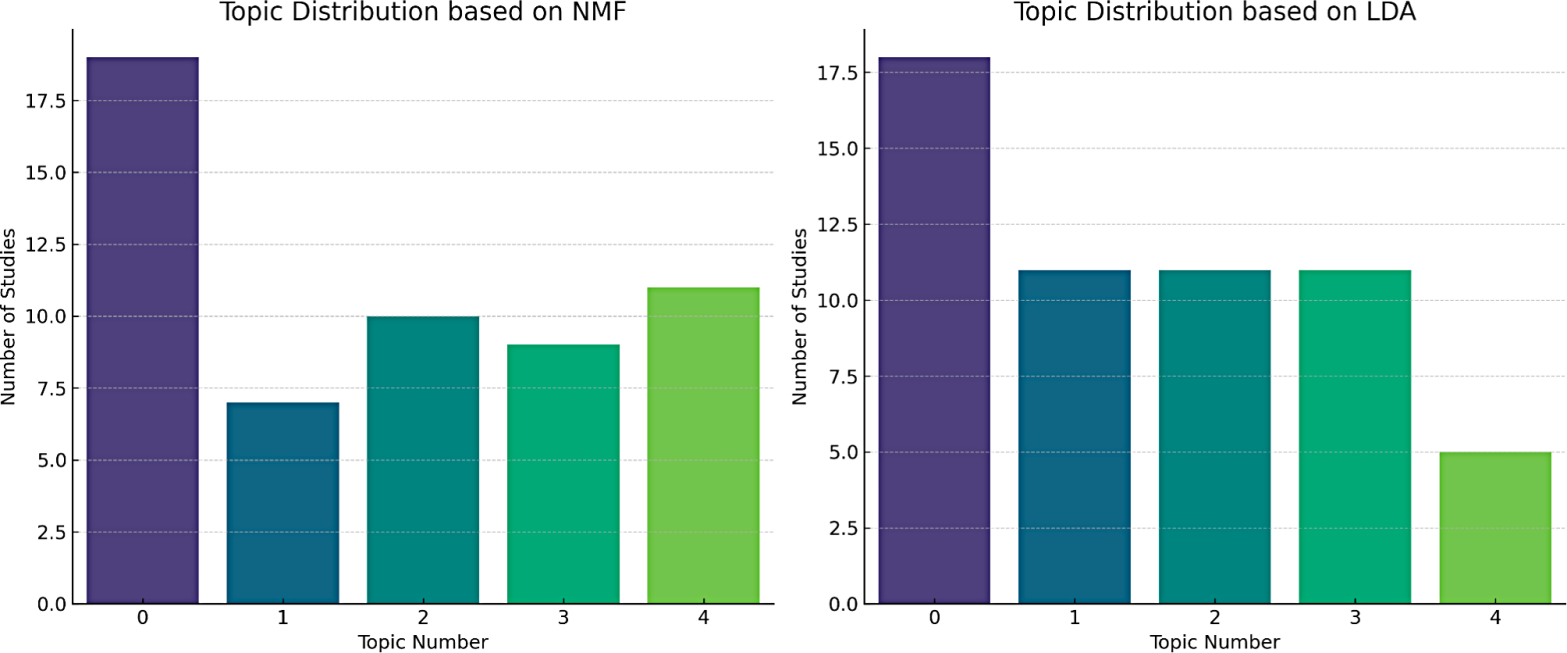
The numerical distribution of the main topics within the key findings of the 56 studies

Based on the results of both the NMF (see SF6) and LDA (see SF4) - i.e., their overlapping emerging themes - the main topics within the key findings of the 56 studies appeared to be the following (see also Fig. 10 below for a numerical distribution):

- **Topic 0: Effects of Music on Infants’ Physiology**: Both NMF and LDA highlight the impact of different types of music (including lullabies and classical music) on infants’ heart rate, respiratory rate, and stress levels. This topic seems to be the most comprehensive, covering physiological responses to music interventions.
- **Topic 1: Pain Management**: Another common topic focuses on how music or sound interventions can alleviate pain, especially during procedures like glucose tests. Terms like ‘pain’ and ‘glucose’ frequently appear in the findings, often in the context of pain scores like PIPP (Premature Infant Pain Profile).
- **Topic 2: Sleep and Stress**: The role of music in affecting sleep quality and reducing cortisol levels in infants is another recurring theme. Both models identify terms related to sleep, stress, and cortisol levels.
- **Topic 3: Feeding and Weight Gain**: This topic centers on the effects of interventions on infants’ feeding habits and weight gain. Terms like ‘weight’, ‘feeding’, and ‘Non-Nutritive Sucking (NNS)’ appear in this context.
- **Topic 4: Methodological Aspects**: While not a ‘finding’ per se, the models also highlighted the frequent mention of study methodologies, with terms like ‘study’, ‘group’, and ‘therapy’ appearing often. This suggests that the dataset contains a variety of research designs and approaches.

**Fig. 11:**
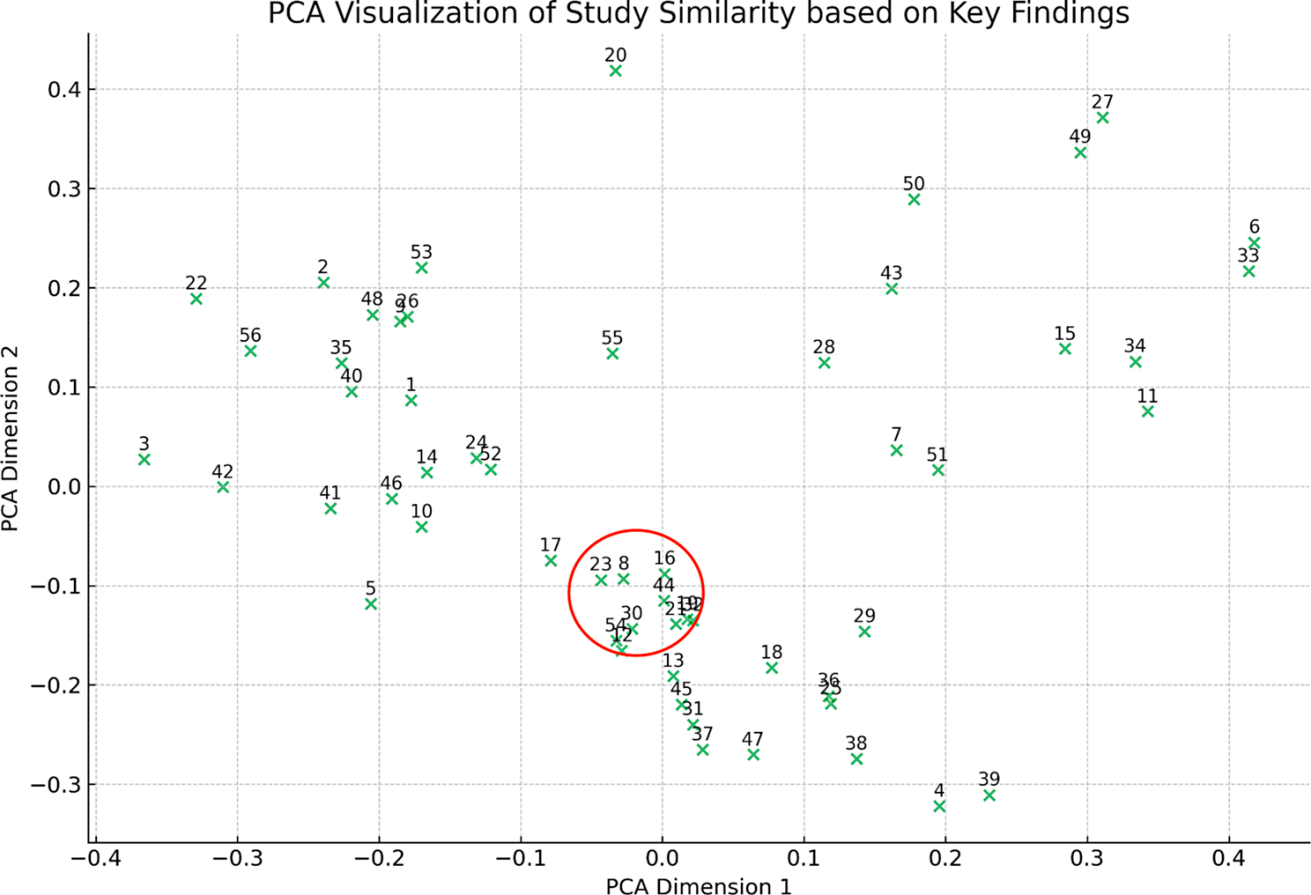
Interrelationships among the main findings of the 56 studies

Finally, the application of the PCA significantly enhanced our understanding of the interrelationships among the key findings of this specific set of studies. By plotting the principal components against each other, we observed distinct clusters within the research landscape. As depicted in Fig. 11, the studies are distributed across the PCA plot, indicating the multidimensionality of the research conducted in this field. Most notably, a particular cluster highlighted in red captured our attention due to its compactness. This cluster comprises studies with Study IDs: [8, 12, 23, 30, 43], which demonstrate a high degree of similarity in terms of their key findings. Such clustering within the PCA plot is indicative of a subset of research that, due to its consistency and potential cumulative evidence, may possess a higher degree of validity or reliability within the broader scope of our review’s thematic focus.

## Discussion

The increasing integration of music into the caregiving environment as a non-pharmacological tool reflects a burgeoning recognition of the music’s potential developmental and therapeutic benefits, while it also highlights its emerging importance in neonatal care. However, despite the growing body of research on this front, inconsistencies and a lack of consensus on the efficacy of music interventions in this context present the need for a more nuanced exploration of the former’s role in neonatal care. For this reason, adhering to PRISMA guidelines, we conducted a comprehensive and systematic literature search specifically on passive music listening, capturing a wide range of interdisciplinary studies until December 2022, ensuring an exhaustive inclusion of relevant literature spanning music therapy, music medicine, clinical care, neonatology, and neurology. In total, 56 articles met our inclusion criteria, while providing a robust dataset to analyze, helping us to address the critical gap in the literature centered around the interplay between theoretical knowledge and practical application in this context. We achieved this by focusing more on the ‘whats’ of this link rather the ‘hows’, following a descriptive statistics comprehensive mapping of the studies’ profiles and methodological structuring, as well as through a synthesis of analytical tools (i.e., Non-negative Matrix Factorization, Latent Dirichlet Allocation, and Principal Component Analysis) applied on the findings of our dataset.

Our first step in this exploration (i.e., the descriptive statistics comprehensive mapping) allowed us to discern overarching patterns and trends within the dataset. Notably, a significant majority of the studies originated from the United States. This geographic concentration necessitates a critical evaluation of potential geographical bias as this US-centric focus of the existing research may influence the applicability and generalizability of these findings on a global scale, potentially skewing the perception of music’s effectiveness and its adaptability in different cultural contexts. Recognizing and addressing this limitation is crucial, as the efficacy and implementation of music in neonatal care might exhibit considerable variations across diverse cultural and geographic settings. By the same token, the deployment of quantitative methods in 82% of the reviewed studies marks a significant trend towards prioritizing empirical data. While this highlights the field’s dedication to empirical rigor, it also reveals a notable gap in qualitative research. The scarcity of qualitative studies is a concern as these methodologies can provide valuable context and insights into personal experiences and outcomes, enriching our understanding beyond what can be quantified, as it is also emphasized in the wider medical literature [33]. The underrepresentation of qualitative approaches may lead to an incomplete portrayal of the nuanced impacts of music therapy and music medicine, aspects that quantitative data alone cannot fully capture. This imbalance underscores the need for an integrative research approach that combines both quantitative and qualitative methods, ensuring a more holistic understanding of the role of music in neonatal care.

Expanding upon this analysis, we most importantly observed a pronounced focus within the research on the application of music in medical treatment and therapy, overshadowing its role in development and prevention. This pattern strongly implies that the immediate concerns of treatment and medical therapy are receiving disproportionate attention within the specific research domain. Conversely, areas related to developmental milestones or procedures, as well as preventative strategies, appear to be less rigorously explored. This discrepancy may signal a prevailing reactive approach in the field, as opposed to a more balanced, proactive strategy that equally values prevention and long-term developmental outcomes. The importance of this finding is also underscored by the current shift in professional medical and clinical bodies advocating for more prevention research and developmental data. For instance, the article by Sabayan et al., [44], titled “Preventive neurology: an emerging field toward brain health” in *Neurology*, reflects this evolving perspective. Additionally, this observation aligns with the findings of Bielenik et al.‘s meta-analysis and review [4], which highlighted a gap in research on long-term outcomes for music therapy with preterm infants.

In conjunction with these observations, our methodological review revealed a striking diversity in data collection methods, ranging from physiological measures to advanced neuroimaging. This diversity exemplifies the field’s openness to various investigative approaches, reflecting a willingness to embrace a range of scientific techniques. However, the broad spectrum of methodologies employed across studies presents a double-edged sword. While this methodological plurality enriches the field, it simultaneously introduces challenges in standardization and comparability of results. The presence of such methodological heterogeneity, as seen in our dataset, underscores the need for consistency in research practices. This finding has been also stressed significantly elsewhere in the same field [28], suggesting that methodological alignment is pivotal for the reliability and replicability of future research, particularly in a field as dynamic and interdisciplinary as music in neonatal care.

Another salient observation from our comprehensive mapping was a strong clinical setting bias, with a significant majority of studies conducted in hospitals. This skew may inadvertently neglect the potential effects of passive music listening in less controlled, everyday environments. Such environments are crucial for a comprehensive understanding of music’s impact on infant development and treatment, especially considering the daily contexts in which children engage with music. Moreover, the representation of professionals within these studies indicated possible regional preferences for certain disciplines, suggesting a need for broader interdisciplinary and geographical parallel engagement as stated above. This aspect is critical to avoid academic insularity and to encourage diverse perspectives, enriching in this way the scope and applicability of research findings.

Finally, a notable gap we found in the literature is the lack of detailed reporting on infants’ pathologies and intervention lengths. This omission hampers the ability to synthesize evidence and draw valid conclusions across studies. Therefore, we believe that addressing this gap is essential for enhancing the comparability and robustness of future scoping reviews and for formulating comprehensive, evidence-based recommendations in the field of neonatal care.

In shifting our focus from descriptive statistics to the analytical outcomes based on the findings of these 56 studies, our investigation transitioned from a broad overview to a more nuanced analysis, revealing pivotal trends and insights into the landscape of passive music listening within neonatal contexts. For example, the dominant exploration of physiological impacts in these studies suggests a strong biological orientation in the field. While this focus aligns with evidence-based practices crucial for safe and effective pediatric care, it may be limiting approaches that address early infant neurobiological and behavioral development in the context of medical stability [48, 49]. Music-based interventions that maximize medical stability within a neurodevelopmental modal may improve developmental trajectories of premature infants and foster greater interdisciplinary collaborations [50, 52].

Moreover, it aids in refining neonatal care protocols, attracting necessary funding, and providing quantifiable outcomes for policy-making and educational initiatives in healthcare. However, this focus may also overshadow the potential contributions of interdisciplinary approaches [7, 51], encompassing psychology, neuroscience, and the arts, which could offer a more comprehensive understanding of infant well-being and development.

Additionally, the overwhelming emphasis on pain management in this context raises ethical considerations regarding the suitability of interventions for this vulnerable group. These considerations include ensuring informed consent and child assent, conducting rigorous risk-benefit analyses, preventing potential exploitation of vulnerable participants, monitoring long-term effects, and respecting cultural sensitivities, elements missing in the studies of this literature review. Such considerations are crucial to safeguard the welfare and rights of child participants in these studies [2], while they also underscore the necessity for heightened ethical discourse and the establishment of more robust guidelines.

Methodologically, the 56 studies exhibit a strong emphasis on rigorous methodology, reflecting the field’s recognition of its importance. However, this focus might also result in a compartmentalization of approaches, possibly at the expense of more innovative and exploratory research as well as specialization of practice. This is not only evident by the fact that most of the 56 studies focus primarily on immediate physiological impacts, therefore revealing a gap in understanding the long-term implications of these interventions. This gap is also indicated by the identification of a monotypic cluster in the Principal Component Analysis (PCA), suggesting that in all of this relevant literature there is only one group of studies heavily reliant on each other, contrary to the expectation of a more diverse range. While this demonstrates a solid line of inquiry for this particular cluster of studies, it could also imply a lack of innovation in both research and practice approaches or subjects, an element that, if evident, may hinder professional development and individual clinical expertise resulting in turn, in suboptimal practice of evidence-based medicine and therapy protocols [34].

On the other hand, this weak but existent centralized approach, ranging mostly from pain management to feeding habits, underscores an emerging potential value of narrative synthesis for clinicians. Yet, this method appears underutilized according to our analysis. This is evident in the fragmented nature of reporting across studies, where findings seem to be presented in isolation, focusing narrowly on specific aspects of infant care such as physiological responses, without considering the broader context or interconnections existing between these or other important areas. For instance, research on the impact of music on pain management in infants is reported separately from studies investigating its effects on feeding behaviors (i.e., no actual links were evident throughout our NMF and LDA analysis), therefore missing, in our view, opportunities to explore potential synergies or holistic impacts of music therapy and music medicine in the wider clinical and domestic context. This lack of integrated perspectives in research is further reflected in the disconnect between research findings and their implementation in public policy. Despite overwhelming support for certain interventions, a discernible gap remains between scientific evidence and policy advocacy.

This discrepancy can be traced back to several nuanced elements within our scoping review. For example, we have seen that the studies predominantly rely on quantitative methods, which, while rigorous, may not adequately capture the complexities and contextual variations essential for shaping effective public policies, as evidenced by the limitations highlighted in quantitative health impact assessments [27]. This methodological inclination towards quantitative data often leads to a gap between what is scientifically validated under controlled conditions and what is practically feasible or relevant in the varied scenarios of public health, thereby hindering the transition of these findings into actionable policies. Compounding this issue is the fact that most of the research is conducted in clinical settings, focusing more narrowly on clinical efficacy. This clinical orientation, however, may not fully address the broader considerations necessary for policy development, such as accessibility, cost-effectiveness, and integration into existing health systems, a concern that is also reflected in other conjoining domains in the same context [9]. Policies require a holistic view that encompasses these wider public health perspectives, which seems to be underrepresented in the current research oeuvre. Additionally, the results indicate a lack of emphasis on translational research and implementation science - that is, studies that bridge the gap between clinical findings and practical application in real-world settings. This absence is significant, as translational research and implementation science are crucial for understanding how to implement research findings effectively and efficiently in diverse healthcare environments; although admittedly its translation into clinical practice is globally slow [1]​​. Without this bridge, a chasm between scientific knowledge and its application in public health policy remains. Lastly, economic evaluations of these interventions are completely lacking, especially concerning the costs and benefits of large-scale implementation. This gap highlights the need for more comprehensive research that encompasses economic aspects, long-term effects, caregiver roles, and policy implications to fully understand and leverage the benefits of music therapy and music medicine in neonatal care, as economic evaluations often remain inaccessible to policymakers [23]​​.

### Methodological limitations

Our scoping review unveils crucial insights into the nuanced effects of passive music listening within the field of medical practices, extending its relevance on infant development. Yet, it is imperative to confront the methodological limitations that pervade our analysis – limitations that may subtly, yet significantly, shape the breadth and reliability of our findings, potentially constraining their applicability and undermining the generalizability of our conclusions.

The most salient limitation, in our point of view, is the intentional exclusion of studies that employed multi-modal or multisensory protocols. By narrowing the focus exclusively to passive music listening, our review may have inadvertently bypassed the broader, and possibly more profound, impact of passive music interventions when combined with other sensory stimuli. Such an exclusion might restrict the scope of our findings, thereby constraining our understanding of music’s role in a more integrated sensory context – a factor that could be particularly pertinent in neonatal care environments.

Moreover, the data selection process presents another layer of complexity. Despite the ostensibly rigorous nature of our methodology – wherein articles were meticulously screened by two independent reviewers, with disputes settled through discussion or, when necessary, the intervention of a third party – this process is not immune to bias. The inherently subjective nature of interpreting eligibility criteria introduces the risk of including or excluding certain studies based on individual discernment, thereby influencing the review’s overall conclusions. Although efforts were made to mitigate such bias, it cannot be entirely eliminated, presenting a crucial, albeit understated, consideration when evaluating the integrity and validity of our outcomes.

Lastly, it is essential to consider the language restriction imposed during the selection of studies. By including only English-language publications—a common practice in scoping reviews—we may have inadvertently introduced a geographical bias. This decision, while necessary to ensure consistency and manageability, could have led to the exclusion of pertinent studies from non-English-speaking regions. Consequently, our findings may not fully reflect the global landscape of research on passive music listening in infant care, thereby limiting the applicability of our conclusions across diverse cultural contexts.

## Conclusion

This scoping review has illuminated the multifaceted role of passive music listening in neonatal care, uncovering both its potential benefits and the gaps in current research. While recognizing the efficacy of music as a therapeutic and developmental tool for infants, there is a compelling need for research that is broader and more culturally diverse, incorporating both qualitative and quantitative methodologies. The future of this field lies in integrating insights from neuroscience with developmental studies, deepening our understanding of how music interacts with the developing brain. Such interdisciplinary research will not only enhance our theoretical knowledge but also improve practical applications in neonatal care worldwide. As we move forward, the challenge is to bridge the gap between scientific understanding and practical application, exploring music’s role beyond clinical settings and into everyday neonatal care. Addressing the ethical dimensions of using music with vulnerable infant populations is also paramount. The aim is to harness the full potential of music therapy and music medicine in a global, culturally nuanced context. By doing so, we can contribute to the holistic well-being and developmental outcomes for children across the globe, ensuring that music becomes a universally accessible and effective tool in neonatal care.

## Electronic supplementary material

Below is the link to the electronic supplementary material.

Supplementary Material 1

Supplementary Material 2

Supplementary Material 3

Supplementary Material 4

Supplementary Material 5

Supplementary Material 6

Supplementary Material 7

Supplementary Material 8

## Data Availability

The datasets used and/or analysed during the current study are available from the corresponding author on reasonable request.
